# Accumulation and Genotoxicity of UiO-66 Nanoparticles in Freshwater Fish *Nothobranchius rachovii*

**DOI:** 10.3390/jox16050175

**Published:** 2026-09-16

**Authors:** Natalia Abramenko, Vadim Vergun, Julia Jukova, Varvara Topolenko, Sergey Simanovsky, Vera Isaeva, Eugene Krysanov, Leonid Kustov

**Affiliations:** 1 A.N. Severtsov Institute of Ecology and Evolution Russian Academy of Sciences, Leninsky Prospect, 33, Moscow 119071, Russia; 2 N.D. Zelinsky Institute of Organic Chemistry Russian Academy of Sciences, Leninsky Prospect, 47, Moscow 119991, Russia; 3 Chemistry Department, Moscow State University, Leninskie Gory 1, bldg. 3, Moscow 119991, Russia

**Keywords:** UiO-66, genotoxicity tests, chromosomal aberration, sister chromatid exchange, accumulation, MOF nanoparticles, cytogenetics

## Abstract

Metal–organic frameworks (MOFs) possess remarkable physicochemical properties, making them highly attractive candidates for advanced drug delivery, catalysis, water purification, gas separation, and biomedical application. Thus, it is important to understand its toxic effect on the environment and human health prior to wide practical application. In this study, UiO-66 nanoparticles (Zr_6_O_4_(OH)_4_—octahedral clusters with benzene-1,4-dicarboxylate linkers) were synthesized and characterized by XRD, SEM, and TEM techniques. The genotoxicity and accumulation of UiO-66 were investigated towards freshwater fish, *Nothobranchius rachovii*, for the first time. To gain a better understanding of the effects of UiO-66 nanoparticles towards *N. rachovii*, fish were exposed to UiO-66, the linker, and zirconium salts. The genotoxic risk caused by UiO-66 was detected by analyzing the Chromosome Aberrations (CAs), frequencies of Sister Chromatid Exchanges (SCEs), mitotic index, and accumulations of Zr in tissues of fish treated with UiO-66 samples, linker, zirconium salt, and non-exposed control subjects.

## 1. Introduction

Anthropogenic advancement has significantly progressed society, yet these achievements have severe effects on ecosystems. Addressing these challenges requires a renewed commitment to environmental research focused on sustainable materials [1]. The use of an efficient nanocarrier for targeted drug delivery systems represents an obvious strategy to improve cancer therapy and diagnostics. Hence, the search for alternative medicinal treatments is a worldwide task. However, synthetic nanocarriers must also be safe and biocompatible to exclude secondary pollution [2,3].

Modern industrial advancement has brought great advantages to humans, but it has also caused severe ecological damage and a negative impact on nature. The enormous number of chemicals produced and applied by humans presents certain risks to the environment due to direct and indirect water contamination. To date, researchers have explored a number of modern techniques to reduce water pollution issues and minimize ecological damage by focusing on environmentally benign materials [3,4].

A new class of porous crystalline materials, metal–organic frameworks (MOFs), demonstrate unique physicochemical properties and show great perspectives for drug delivery, catalysis, wastewater treatment, gas separation, and biomedical applications. In this context, UiO-66 material, which is constructed of [Zr_6_O_4_(OH)_4_]^12+^ octahedral clusters with benzene-1,4-dicarboxylate (bdc) ligands. UiO-66 has received considerable attention for environmental remediation and drug delivery systems because of its outstanding physicochemical properties, including chemical and thermal stability, high porosity [5], biocompatibility, and low toxicity [6,7,8]. The nano-sized UiO-66 may have potential biomedical application in drug delivery systems, medical diagnostics, bio-sensing, bioelectronics, gene therapy, and imaging [9,10,11,12].

Several works have reported the medical application of the UiO-66 nanoparticles (NPs) as carriers in drug delivery systems and cancer therapy [13,14,15,16,17,18]. The composite with UiO-66 exhibits pH-sensitive behavior and might be applied as an effective nanocarrier for targeted drug delivery systems of pantoprazole [17]. The UiO-66 nanomaterials were successfully applied as photosensitizing agents in cancer therapy [10]. For instance, the composite based on UiO-66 NPs and polyaniline demonstrated a significant photothermal therapeutic effect in vitro and effective inhibition of the growth of colon cancers in vivo [19]. Composites of porphyrin derivatives and nano-MOF were considered as effective materials for photodynamic therapy of resistant head and neck cancer [20]. The promising prospects and potential wide applications of UiO-66 can lead to its leaking into the environment. UiO-66 can enter the aquatic environment as part of wastewater treatment, through industrial discharges, and via improper disposal. Considering the expanding potential for applications of UiO-66, it is vital to assess its safety profile and impact on nature.

Several studies have observed the toxicity and compatibility of nano-MOFs in vitro [21], and even more rarely in vivo. It was shown that the cytotoxicity of MOF nanomaterials strongly depends not only on the MOF composition but also on the cell type [22,23].

There are several scientific papers where the genotoxicity of zirconium compounds and Zr-containing NPs were investigated [24,25,26,27]. For instance, Atalay et al. [28] examined genotoxic and apoptotic effects of ZrO_2_ NPs on fibroblast cells. It was shown that ZrO_2_ NPs caused significant DNA damage and apoptotic effects on L929 cells. Clastogenic effects of zirconium oxychloride were demonstrated towards bone marrow cells of mice in vivo [29]. The frequencies of chromosomal breaks and alterations induced by zirconium oxychloride increased significantly in a dose-dependent manner. A similar effect of zirconium oxychloride was demonstrated for human peripheral blood lymphocytes [30]. The aberrations increased significantly when compared with their respective untreated cells. Reports on UiO-66 NPs toxicity and specific medicinal properties are very limited. Moreover, to the best of our knowledge, there are no examples in the literature of investigations of the potential genotoxicity of UiO-66 nanomaterials.

In this study, we evaluated the genotoxicity of UiO-66 NPs towards African annual killifish *Nothobranchius rachovii* (*N. rachovii*), a convenient model for laboratory studies of fish genotoxicity [31,32]. Its key advantages include one of the lowest diploid chromosome numbers among fish (2n = 16), simplicity of laboratory breeding, and a rapid 4–6-month life cycle with sexual maturity reached in 3–4 weeks. High mitotic activity in the pronephros ensures abundant, high-quality metaphase plates containing a small number of large chromosomes, simplifying cytogenetic analysis.

The influence of UiO-66 NPs on the Chromosome Aberrations (CAs), frequencies of Sister Chromatid Exchanges (SCEs), and mitotic index were analyzed as end points. Accumulation of zirconium in *N. rachovii* organs was investigated after intraperitoneal injection. Toxicity of UiO-66 NPs was compared with the data for Zr^4+^ salts and linker. Eventually, the findings of this research provide essential empirical assistance to understand the genotoxic and clastogenic effect of UiO-66 NPs and offer a scientific basis for their safe, rational application.

## 2. Materials and Methods

### 2.1. Chemicals

ZrCl_4_ (99+%, Sigma-Aldrich, St. Louis, MO, USA), ZrOCl_2_ × 8H_2_O (99+%, Sigma-Aldrich, St. Louis, MO, USA), H_2_bdc (99+%, Sigma-Aldrich), potassium chloride (99+%, Sigma-Aldrich, St. Louis, MO, USA), methanol (≥99.8%, Sigma-Aldrich, St. Louis, MO, USA), acetic acid (30%, Sigma-Aldrich, St. Louis, MO, USA), hydrochloric acid (37%, Sigma-Aldrich, St. Louis, MO, USA), phosphate-buffered saline (Sigma-Aldrich, St. Louis, MO, USA), 5-bromo-2-deoxyuridine (>97%, Sigma-Aldrich, St. Louis, MO, USA), Giemsa stain (Sigma-Aldrich, St. Louis, MO, USA), and Hoechst 33258 (98%, Fisher Scientific, Schwerte, Germany) were used in the experiments.

### 2.2. Synthesis and Characterization of UiO-66 Materials

UiO-66 sample was synthesized by modified synthesis [33] and were characterized by powder X-ray diffraction and electron microscopy measurements [34]. (Supplementary Materials).

### 2.3. Ecotoxicity Testing

In vivo Chromosomal aberration and SCE assay. The mitotic index assessment.

Laboratory stock of African annual killifish *Nothobranchius rachovii* (Cyprinodontiformes) were maintained in aquaria with weak aeration under a 14 day/10 nights cycle [31,32]. The fish were fed with *Artemia nauplii* daily. All animal experiments were approved by the A.N. Severtsov Institute of Ecology and Evolution Animal Ethics Committee (protocol No. 108, 25 October 2024).

The fish were kept in a synthetic medium with the composition 50 mM NaCl, 1.7 mM KCl, 3.3 mM CaCl_2_, and 3.3 mM MgSO_4_ throughout the experiment. Individuals of *N. rachovii* were injected intraperitoneally with 25 µL each of UiO-66, ZrOCl_2_ × 8H_2_O, ZrCl_4,_ H_2_bdc linker, and distilled water (the concentration of Zr^4+^ ions was ~3 mg/L based on the previous work with zirconium oxide NPs [26,27]). Eight fish (4 males and 4 females) aged 2.5 months were used for each reagent. The individuals were placed in 10-liter aquaria with weak aeration, t = 26 °C, pH = 6.8–7.0, light regime 14 day/10 nights, 96 h. After 72 h, 5 bromo-2-deoxyuridine (BrdU) at a concentration of 100 mg/L was added to the aquaria for 48 h. The fish were then injected intraperitoneally with a 0.1% colchicine solution for 3 h. Next, the pronephros was removed from the fish after euthanasia with a MS-222 solution, hypotonic treated in a 0.075 M potassium chloride solution and fixed in a mixture of methanol and glacial acetic acid (3:1). Metaphase preparations were obtained according to the method described elsewhere [35]. Staining was then performed to detect sister chromatid exchanges according to [36]. Preparations were analyzed with an Axioplan Zeiss microscope at 100× magnification (Carl Zeiss Microscopy GmbH, Jena, Germany). At least 50 metaphases from each fish were analyzed to count chromosomal aberrations, and at least 30 metaphases were analyzed to count sister chromatid exchanges. The mitotic index calculated using the following formula: Mitotic index = (Number of cells in mitosis/Total number of cells) × 100.

### 2.4. Elemental Analysis

Further research was focused on accumulation of Zr in the main internal organs of the fish. The samples of the tissues for the analysis were taken following intraperitoneal injection after 1 day of exposure. Accumulation of Zr was assessed in the gastrointestinal tract (GI), liver, brain, spleen, gonads, and pronephros. A total of forty-eight tissue samples were collected from tested fish.

Elemental qualitative and quantitative analysis of collected samples was carried out in accordance with ISO/TS 18507:2015 guideline [37]. The tissue sample was dissolved in nitric acid (1:1) in a MARS high-pressure microwave oven (CEM Corporation, USA), then suspended in 1% Triton X-100 along with the internal standard. A standard gallium nitrate solution (GSO 7641-98, GDVI.410408.024.PS) was introduced into each tested sample at 1 mg/L of the initial liquid. The obtained mixture was mounted onto special quartz disks and dried at room temperature, after the probe was placed in the spectrometer. Elemental analysis was measured by using a Rigaku NANOHUNTER spectrometer (Rigaku Corporation, Tokyo, Japan) by total external reflection X-ray method.

Statistical analysis of the results was carried out using the program GraphPad Prism version 8.0 (GraphPad Software, San Diego, CA, USA) and IBM SPSS v. 27 (New York, NY, USA). All data were presented as mean ± standard deviation. The *t*-test, 2-way ANOVA, and Mann–Whitney U test were used for significance analysis. The significant differences were set as * *p <* 0.05.

## 3. Results and Discussion

### 3.1. Characterization of the UiO-66 Nanomaterials

**Figure 1:**
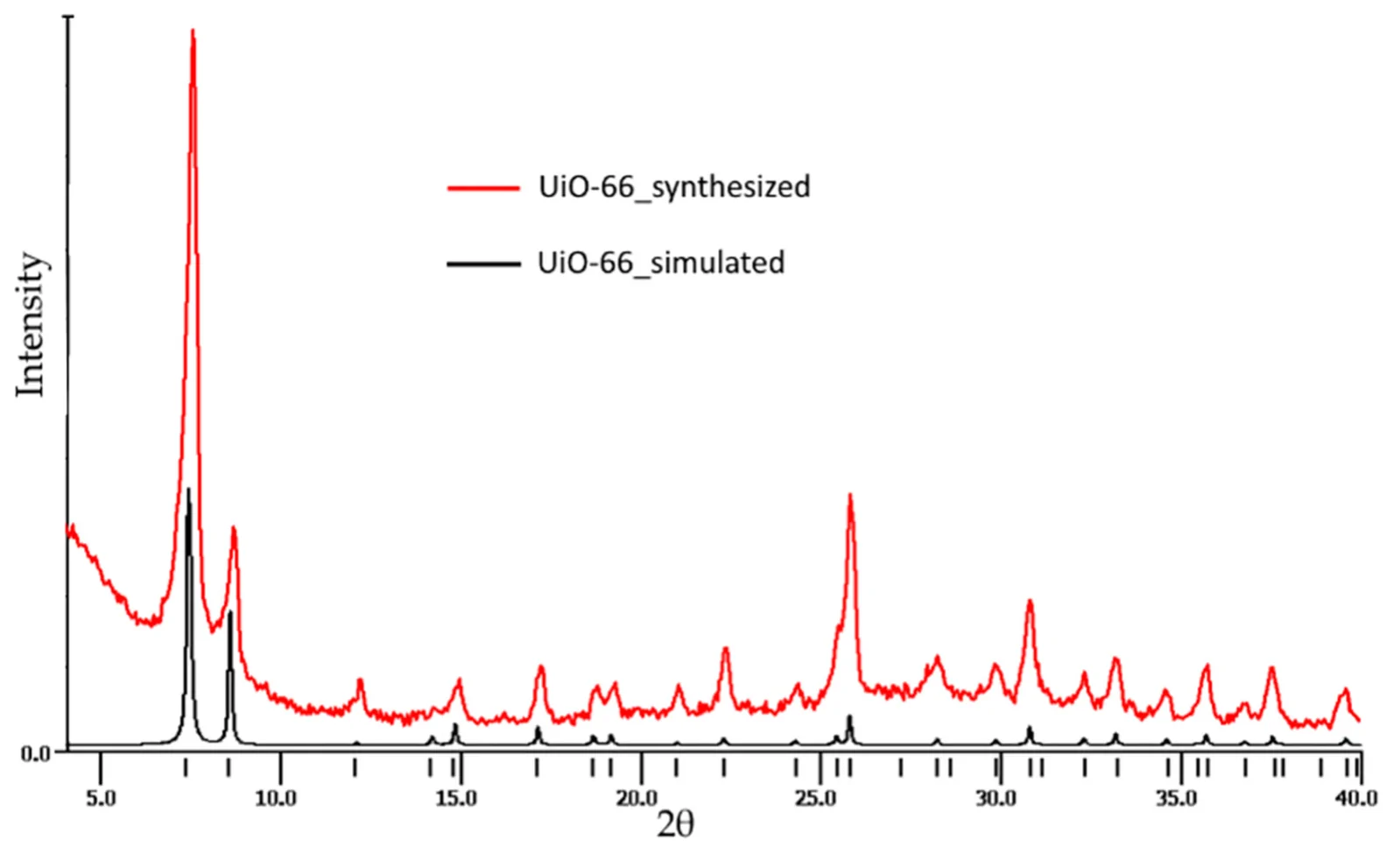
PXRD pattern of the UiO-66 sample.

The structural characteristics of the prepared UiO-66 powder were evaluated by powder X-ray diffraction data (Figure 1).

Synthesized UiO-66 samples correspond to the crystalline UiO-66 structure with a cubic symmetry (structural type Fm-3m); the sample contains the only phase. Abundant mesopores should be assumed in the UiO-66 nanomaterials by analysis of their XRD patterns with a broad peak in the region of 2–4 2Θ.

**Figure 2:**
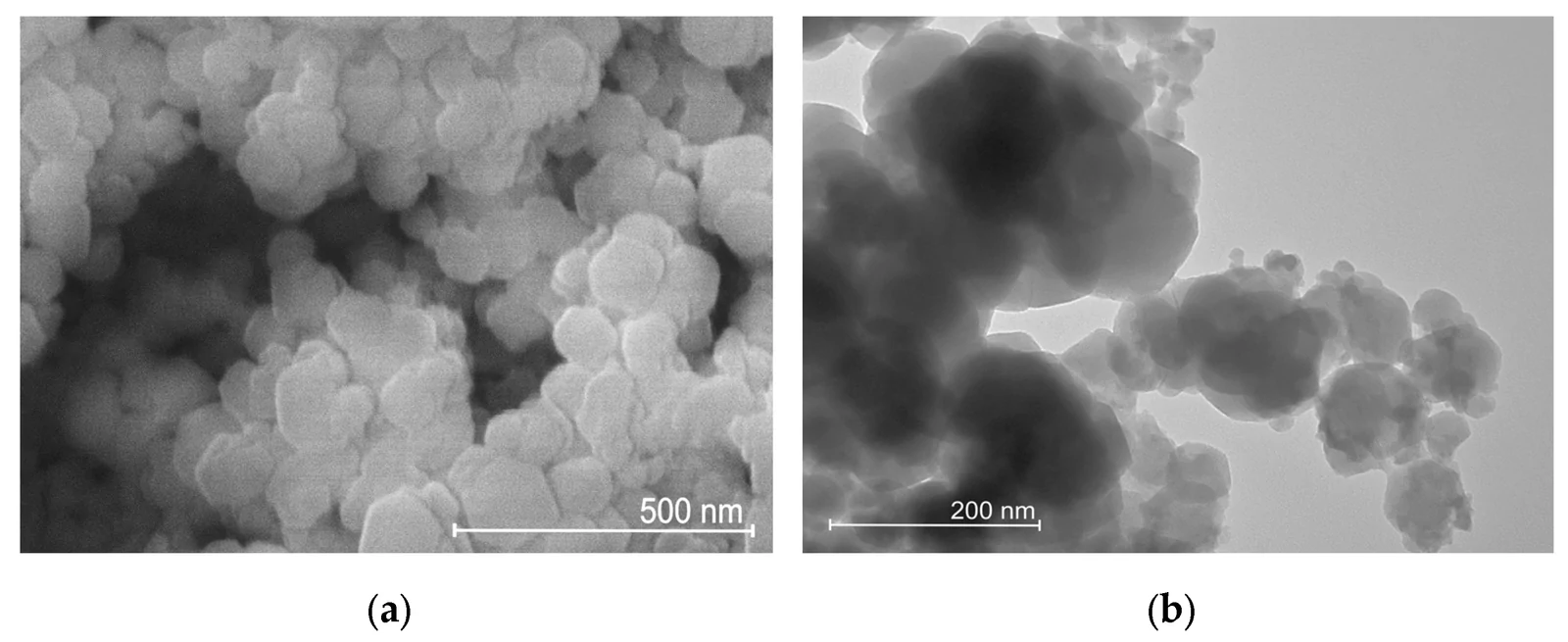
SEM (**a**), TEM (**b**) images UiO-66 sample.

The UiO-66 sample consists of 78 ± 23 nm (average size and standard deviation) size particles, the particle shape is distorted spherical, the octahedron faces are almost invisible (Figure 2). The sample UiO-66 shows crystallite organization into rather large agglomerates. Note that this small NP size of the produced UiO-66 material allows one to exploit it in biomedical applications.

### 3.2. Toxicity Tests

In this study, we observed the hazard effect of UiO-66 NPs using *N. rachovii*. Chromosome Aberrations (CAs) and frequencies of Sister Chromatid Exchanges (SCEs) were estimated in the pronephros tissue for fish treated by UiO-66 sample, linker, Zr salts, and for non-exposed control subjects. The results of frequencies of CAs are summarized in Table 1.

In general, the frequencies of aberrations were similar for UiO-66 sample and ZrCl_4_ to the results in the control groups. Changes in CAs induced by UiO-66 and zirconium chloride were not significant. Thus, UiO-66 NPs did not cause any clastogenic effect on *N.rachovii* pronephros cells in vivo. Our data are consistent with the results of studies that observed little or no toxicity of UiO-66 in vivo in fish (*Danio rerio*) and in vitro in cell cultures (*HepG2* and *MCF7* cells) [22]. In a study by Yang et al. [38], UiO-66 NPs were non-toxic to adult zebrafish. According to our results of frequencies of CAs, we found that zirconium oxychloride had a higher toxic effect towards fish in comparison with UiO-66. The observed effect with respect to frequencies of CAs was determined predominantly by the anion (oxychloride). A similar significant effect induced by zirconium oxychloride for chromosomal aberrations was observed in bone marrow cells of mice in vivo [29]. Evidently, zirconium ions or UiO-66 do not demonstrate any significant genotoxic effect in *N. rachovii* pronephros cells.

In order to evaluate the level of the potential genomic damage caused by UiO-66 NPs, cytogenetic analyses were performed by using SCE and MI assay with *N. rachovii*. Frequencies of SCE and mitotic index data are summarized in Table 2.

**Figure 3:**
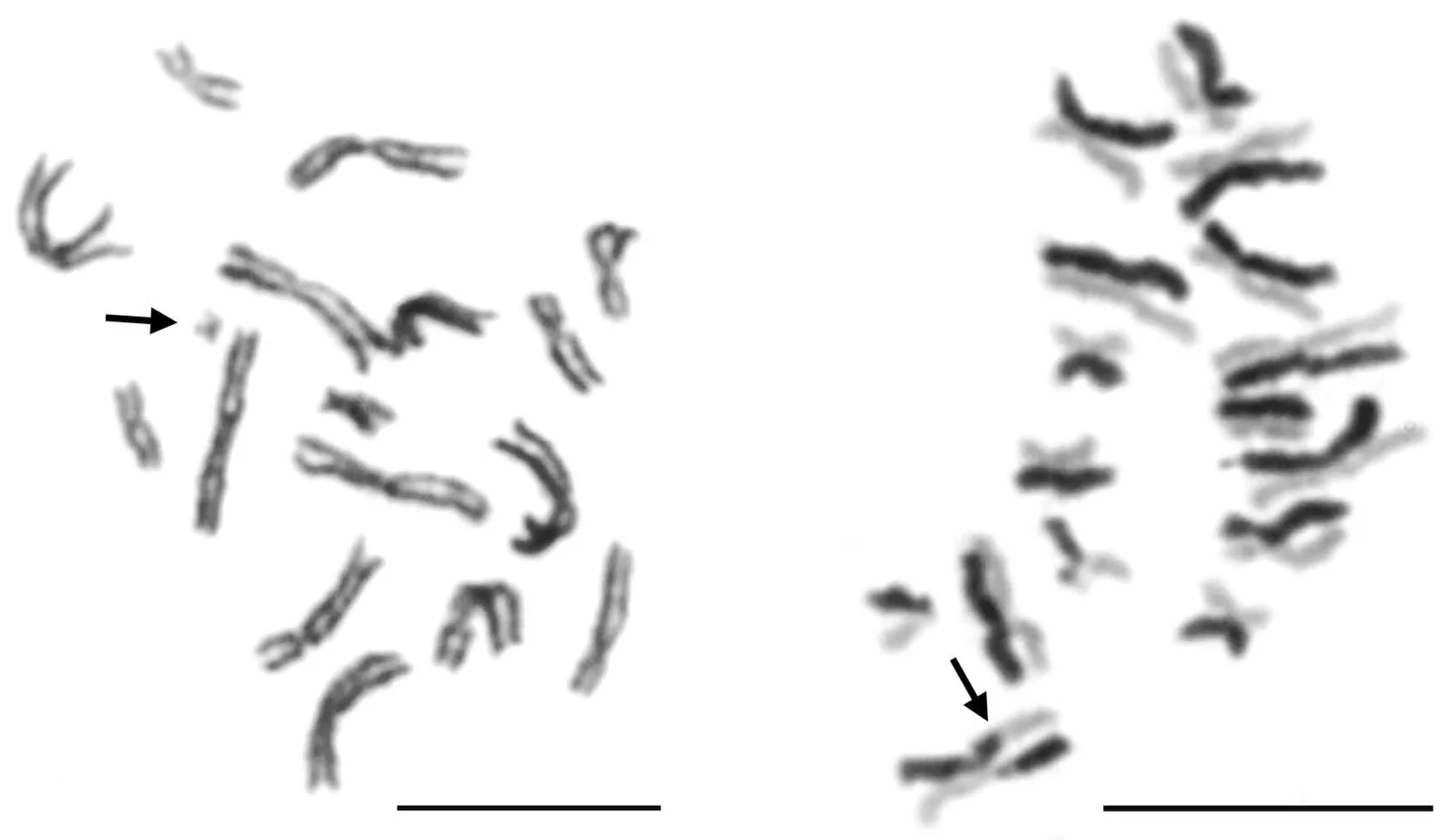
(**a**) Aberrant metaphase from *N. rachovii* pronephros cells (arrow indicate chromosome fragment), (**b**) metaphase from *N. rachovii* pronephros cells with sister chromatid differentiation (arrow indicates sister chromatid exchange). Bars = 10 µm.

UiO-66 and zirconium ions did not induce significant changes in MI. Comparatively, analysis of SCE frequencies showed that all tested compounds significantly increased their frequencies compared to the control, indicating a genotoxic effect that may reflect a potential for genomic instability. The differences in the results of CA, MI, and SCE data may be due to the various sensitivity and focus of applied methods. The method of SCE counting belongs to sensitive methods, the resolution of which is significantly higher than that of the chromosomal aberration counting method. Figure 3 shows aberrant metaphase from *N. rachovii* pronephros cells. The main types of CAs were chromatid and chromosome breaks.

**Figure 4:**
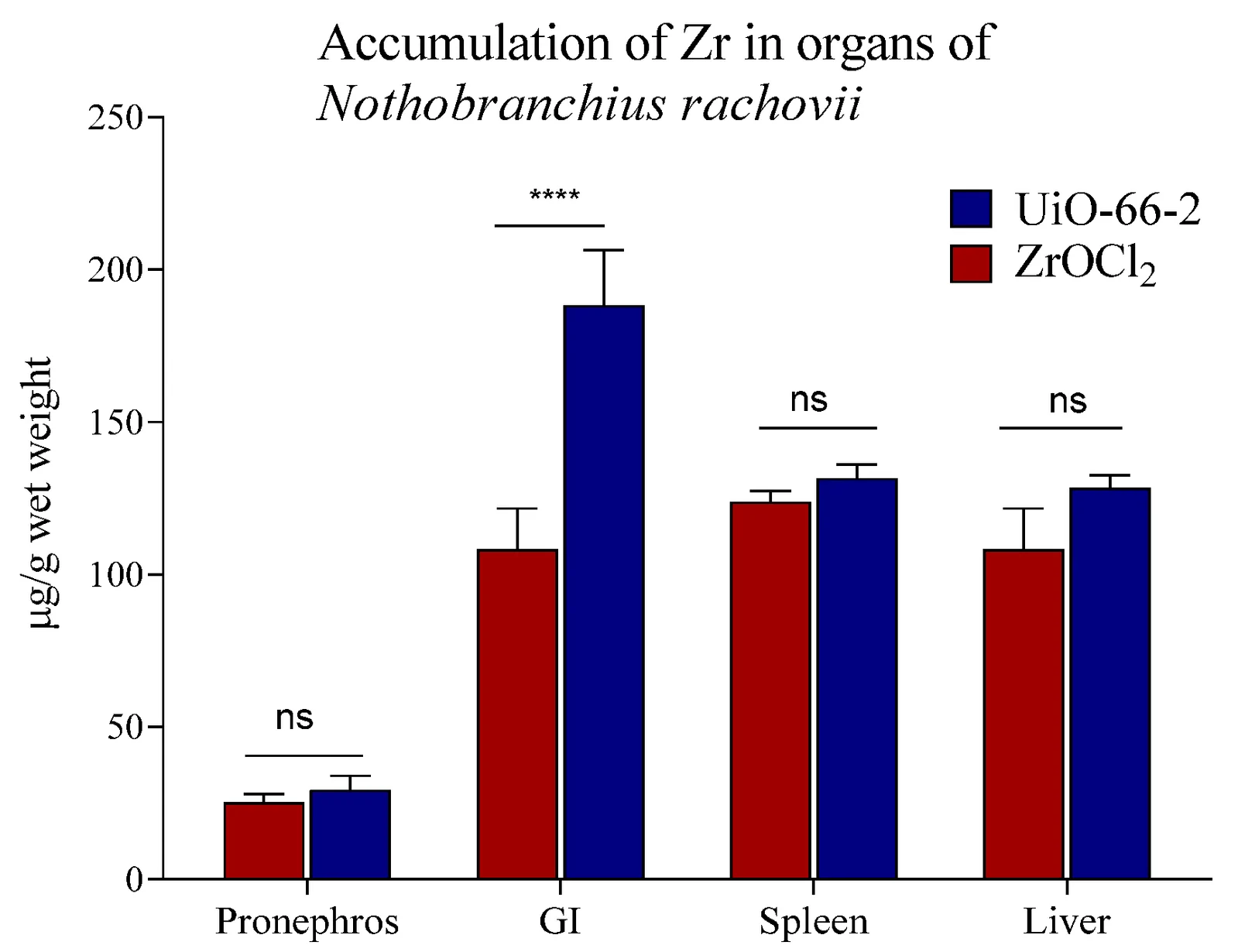
Accumulation of zirconium in the organs of fish *N. rachovii* (mean and standard deviation). In control groups, fish were injected with 25 µL of distilled water, all fish in the control groups were alive at the end of experiments (****—statistically significant; ns—statistically not significant).

The next part of the experiment was related to the study of bioaccumulation of zirconium in *N. rachovii* organs. The toxic effect of the UiO-66 nanomaterials was evaluated by testing the Zr accumulation in the tissues of fish after intraperitoneal administration of 25 µL of UiO-66 and ZrOCl_2_ × 8H_2_O. Accumulation of Zr was measured in the gastrointestinal tract (GI), spleen, liver, and pronephros of tested and control groups of fish. The data are presented in Figure 4.

The maximum amount of accumulated zirconium from UiO-66 NPs was observed in the GI tract of fish. The distribution of zirconium among organs was following: pronephros < liver < spleen < GI tract. Accumulation of zirconium in the brain and gonad samples were below the limit of detection. According to results, Zr species from UiO-66 and salt have shown similar distribution in organs with the exception for the GI tract. Slight differences were revealed in the amount of zirconium in organs depending on the type of material. The higher accumulation of Zr in GI tract aligns with recent in vivo findings for *Daphnia magna* [39]. At the same time, it was demonstrated that UiO-66 NPs, which are safe for zebrafish, may alter the dominant microorganisms in the fish intestine and lead to an increase in microbial markers of intestinal inflammation [38].

UiO-66 is considered as safe and effective porous materials for medical and remediation purposes [40]. The structural and chemical transformations of UiO-66 are a critical factor for its medical application. Although zirconium-based MOFs exhibit pronounced structural stability and strong metal–ligand bonding—with UiO-66, in particular, demonstrating a relatively slow degradation rate under various conditions (a wide pH range, several solvents, and physiological conditions) [41,42]—this pronounced structural stability raises questions regarding the risks of chronic and secondary toxicity. Simultaneously, UiO-66 is sensitive to the presence of electrophilic cationic species and to biotic factors [39]. For instance, using chlorosulfonic acid as a sulfonating agent can cause the decomposition of its crystalline structure [42]. Moreover, a recent study investigated a transformation for UiO-66 in different media found and that it remains stable to atmospheric and aqueous challenges, but it is highly vulnerable to biotic reprocessing [39]. The authors suggested that during digestion, the terephthalate linkers are displaced or degraded from the UiO-66 structure, causing the Zr nodes to reprecipitate within the complex matrix of the gut contents and microbiome. Evidentially, the remediation application of stable UiO-66 can cause zirconium ions to persist in the environment for extended periods, leading to its potential bioaccumulation in aquatic species over time [39]. A recent research showed that UiO-66, at concentrations commonly used in remediation, may lead to algal physiological damage [43]. Thereby, systematic studies of the safety profiles of UiO-66 are necessary, and its environmental stability requires further investigation.

## 4. Conclusions

UiO-66 is typically characterized by a high degree of stability and minor degradation under experimental conditions. The biocompatibility and low toxicity of UiO-66 enhance its potential as a dual-purpose platform that bridges advanced medicine and environmental remediation. This work contributed to an assessment of the prospects of the biomedical applications of UiO-66-based nanomaterials.

In this study, genotoxic effects of UiO-66 NPs in *N. rachovii* were evaluated using the CAs and SCE methods in pronephros cells of fish. To the best of our knowledge, this is the first scientific report on the genotoxic effect of UiO-66 materials. No alterations were observed in CAs and MI caused by UiO-66 NPs. According to elevated SCE results, UiO-66 NPs might cause weak genotoxic potential. For UiO-66, the toxic effect may be related to structural defects and degradation under the experimental conditions and biotransformation in live species. According to the obtained results, UiO-66 does not show substantial risk on pronephros cells of fish at the cytogenetic level.

The observed distribution of zirconium among the organs of fish tissues indicates that zirconium can transfer and accumulate in the fish body after intraperitoneal injection. Taken together, our results and recent experimental data underscore the critical importance of further studies to evaluate the long-term environmental behavior and toxicity of UiO-66 within realistic matrices. Future research should specifically focus on organism-level biotransformation, long-term effects, and toxicity on various levels for different types of species.

## Figures and Tables

**Table 1:** Frequencies of chromosome aberrations in *N. rachovii* pronephros cells.

Samples	Aberrant Cells (Mean ± SD) %
UiO-66	1.03 ± 2.04
ZrCl_4_	2.31 ± 1.60
ZrOCl_2_ × 8H_2_O	5.27 ± 5.35 *
linker H_2_bdc	0.00 ± 0.00
control	1.31 ± 0.30

* The significant differences were set as * *p* < 0.05 (Mann–Whitney test).

**Table 2:** The effect of UiO-66 on frequencies of SCE and MI (metaphases) in *N. rachovii* pronephros cells.

Samples	SCE ± SD%	MI (Metaphases) ± SD %
UiO-66	2.23 ± 0.49 *	0.03 ± 0.02
ZrOCl_2_ × 8H_2_O	1.97 ± 0.48 *	0.08 ± 0.08
linker H_2_bdc	1.17 ± 0.29 *	0.22 ± 0.13 *
control	0.73 ± 0.17	0.05 ± 0.05

* The significant differences were set as * *p* < 0.05 (Mann–Whitney test).

## Data Availability

The original contributions presented in this study are included in the article/Supplementary Materials. Further inquiries can be directed to the corresponding author.

## Supplementary Materials

The following supporting information can be downloaded at https://www.mdpi.com/article/10.3390/jox16050175/s1, synthesis and characterization of tested UiO-66 NPs.
