# Supplementary material for: Accumulation and Genotoxicity of UiO-66 Nanoparticles in Freshwater Fish Nothobranchius rachovii
Source: J Xenobiot. 2026 Sep 16;16(5):175. doi: 10.3390/jox16050175 (PMC13608222; doi:10.3390/jox16050175)
Supplement: Supplementary file 1 [file jox-16-00175-s001.zip › jox-4511341-supplementary.pdf]

Supplementary Materials: Accumulation and Genotoxicity of UiO-66 Nanoparticles in Freshwater Fish Nothobranchius rachovii

Natalia Abramenko, Vadim Vergun, Julia Jukova, Varvara Topolenko, Sergey Simanovsky, Vera Isaeva, Eugene Krysanov and Leonid Kustov

Materials and Methods

Chemicals

$ZrCl_4$ (99+%, Sigma-Aldrich, St. Louis, MO, USA), $ZrOCl_2 \times 8H_2O$ (99+%, Sigma-Aldrich, St. Louis, MO, USA), H_2bdc (99+%, Sigma-Aldrich, St. Louis, MO, USA), potassium chloride (99+%, Sigma-Aldrich, St. Louis, MO, USA), acetone (≥99.5% Sigma-Aldrich, St. Louis, MO, USA), methanol (≥99.8%, Sigma-Aldrich, St. Louis, MO, USA), N,N′-dimethylformamide (DMF, 99.8% Thermo Fisher Scientific, Geel, Belgium, EU) acetic acid (30%, Sigma-Aldrich, St. Louis, MO, USA), hydrochloric acid (37%, Sigma-Aldrich, St. Louis, MO, USA), phosphate buffered saline (Sigma-Aldrich, St. Louis, MO, USA), 5-bromo-2-deoxyuridine (>97%, Sigma-Aldrich, St. Louis, MO, USA), Giemsa stain, Hoechst 33258 (98 %, Fisher Scientific， Schwerte, Germany) were used in the experiments.

Synthesis and characterization of UiO-66 materials

UiO-66 nanomaterial was synthesized by modified procedure [1]. $ZrCl_4$ (233 mg, 1 mmol) and benzene-1,4-dicarboxylate (H_2bdc, 166 mg, 1 mmol) were dissolved separately in two acetone portions (90 mL). The obtained solutions were combined and transferred into an autoclave reactor for 24 h at 80 °C. The synthesized white precipitate was washed with acetone (2 × 20 mL), DMF (2 × 20 mL), and methanol (2 × 20 mL), then activated under a vacuum (8 h, 150 °C, 10-3 Torr). The product yield was 225 mg (~ 81.2%).

Electron microscopy measurements.

A target-oriented approach was applied for the optimization of the analytical meaurements [2]. Before measurements, the sample was mounted on a 25 mm aluminum specimen stub, fixed by a conductive carbon tape and coated with a 30 nm carbon film. The observations were carried out using a Hitachi Regulus 8230 field-emission scanning electron microscope (FE-SEM, Hitachi High-Tech America, Inc., Schaumburg, IL, USA). Images were acquired in a backscattered electron mode (compositional contrast) at a 5 kV accelerating voltage and in secondary electrons at a 5 kV accelerating voltage. For TEM measurements of the samples deposited on 3 mm carbon coated copper grids from an isopropanol suspension were carried out. The morphology of the samples was studied using a Hitachi HT7700 transmission electron microscope (Hitachi High-Tech America, Inc., Schaumburg, IL, USA). Images were acquired in a bright-field TEM mode at a 100kV accelerating voltage.

References

1. Lozano, L.A.; Iglesias, C.M.; Faroldi, B.M.C.; Ulla, M.A. and Zamaro, J.M. Efficient solvothermal synthesis of highly porous UiO-66 nanocrystals in dimethylformamide-free media. *J. Mater. Sci.* **2018**, *53*, 1862–1873. https://doi.org/10.1007/s10853-017-1658-5
2. Kachala, V.V.; Khemchyan, L.L.; Kashin, A.A.; Orlov, N.N.; Grachev, A.A.; Zalesskiy, S.S. and Ananikov, V.P. Target-oriented analysis of gaseous, liquid and solid chemical systems by mass spectrometry, nuclear magnetic resonance spectroscopy and electron microscopy. *Russ. Chem. Rev.*, **2013**, *82*, 648–685. https://doi.org/10.1070/RC2013v082n07ABEH004413.
